# Phosphoglycerate Mutase 1 Prevents Neuronal Death from Ischemic Damage by Reducing Neuroinflammation in the Rabbit Spinal Cord

**DOI:** 10.3390/ijms21197425

**Published:** 2020-10-08

**Authors:** Hyo Young Jung, Hyun Jung Kwon, Woosuk Kim, Kyu Ri Hahn, Seung Myung Moon, Yeo Sung Yoon, Dae Won Kim, In Koo Hwang

**Affiliations:** 1 Department of Anatomy and Cell Biology, College of Veterinary Medicine, and Research Institute for Veterinary Science, Seoul National University, Seoul 08826, Korea; hyoyoung@snu.ac.kr (H.Y.J.); tank3430@hallym.ac.kr (W.K.); hkinging@snu.ac.kr (K.R.H.); ysyoon@snu.ac.kr (Y.S.Y.); 2 Department of Biochemistry and Molecular Biology, Research Institute of Oral Sciences, College of Dentistry, Gangneung-Wonju National University, Gangneung 25457, Korea; donuts25@gwnu.ac.kr; 3 Department of Biomedical Sciences, and Research Institute for Bioscience and Biotechnology, Hallym University, Chuncheon 24252, Korea; 4 Department of Neurosurgery, Dongtan Sacred Heart Hospital, College of Medicine, Hallym University, Hwaseong 18450, Korea; nsmsm@hallym.ac.kr; 5 Research Institute for Complementary & Alternative Medicine, Hallym University, Chuncheon 24253, Korea

**Keywords:** phosphoglycerate mutase 1, ischemia, spinal cord, oxidative stress, pro-inflammatory cytokine

## Abstract

Phosphoglycerate mutase 1 (PGAM1) is a glycolytic enzyme that increases glycolytic flux in the brain. In the present study, we examined the effects of PGAM1 in conditions of oxidative stress and ischemic damage in motor neuron-like (NSC34) cells and the rabbit spinal cord. A Tat-PGAM1 fusion protein was prepared to allow easy crossing of the blood-brain barrier, and Control-PGAM1 was synthesized without the Tat peptide protein transduction domain. Intracellular delivery of Tat-PGAM1, not Control-PGAM1, was achieved in a time- and concentration-dependent manner. Immunofluorescent staining confirmed the intracellular expression of Tat-PGAM1 in NSC34 cells. Tat-PGAM1, but not Control-PGAM1, significantly alleviated H_2_O_2_-induced oxidative stress, neuronal death, mitogen-activated protein kinase, and apoptosis-inducing factor expression in NSC34 cells. After ischemia induction in the spinal cord, Tat-PGAM1 treatment significantly improved ischemia-induced neurological impairments and ameliorated neuronal cell death in the ventral horn of the spinal cord 72 h after ischemia. Tat-PGAM1 treatment significantly mitigated the ischemia-induced increase in malondialdehyde and 8-iso-prostaglandin F2α production in the spinal cord. In addition, Tat-PGAM1, but not Control-PGAM1, significantly decreased microglial activation and secretion of pro-inflammatory cytokines, such as interleukin (IL)-1β, IL-6, and tumor necrosis factor (TNF)-α induced by ischemia in the ventral horn of the spinal cord. These results suggest that Tat-PGAM1 can be used as a therapeutic agent to reduce spinal cord ischemia-induced neuronal damage by lowering the oxidative stress, microglial activation, and secretion of pro-inflammatory cytokines, such as IL-1β, IL-6, and TNF-α.

## 1. Introduction

Spinal cord ischemia is a subject of high interest in the field of neuroscience, and the current research focuses on understanding the pathophysiology of neuronal death after ischemia and identifying preventive or therapeutic agents to overcome ischemia-related neuronal damage [1,2]. Clinically, spinal cord ischemia and subsequent neuronal damage can be predicted, because interruption of the abdominal aorta is a medical requirement in cases of total en bloc spondylectomy and aortic repair surgery, and spinal cord ischemia occurs only in 0.3–1% of all strokes [3]. Spinal cord ischemia is also associated with thoracoabdominal aortic aneurysm [4]. Several potential mechanisms underlying these events have been identified, such as energy depletion, oxidative stress, and the activation of inflammatory pathways after spinal cord ischemia [1,5,6,7,8].

The initial step in energy production from glucose is glycolysis. This process is controlled by phosphoglycerate mutase (PGAM) [E.C. 5.4.2.1], which catalyzes the interconversion of 2- and 3-phosphoglycerate [9,10]. In mammals, three isozymes (mm-, mb-, or bb-type) are present, classified based on the subunits of the PGAM dimer. Among these isozymes, mm- and bb-type isozymes are ubiquitously expressed in most tissues, including the brain, and mm- and mb-type isozymes are expressed in smooth, cardiac, and skeletal muscles. The brain form of PGAM (PGAM1) increases glycolysis in the brain and facilitates ATP production; however, oxidative modification inhibits glycolysis and, eventually, depletes ATP in the brain [11]. 

PGAM1 is overexpressed in several types of cancer cells in humans and is related to the growth, survival, and invasion of tumors [12]. In addition, PGAM1 can be a promising target for diagnosis and therapy in tumoral cells [12]. The ectopic expression of PGAM1 was shown to decrease senescence-induced oxidative damage in a primary mouse embryonic fibroblast cell line [13]. In fibroblasts, PGAM1 levels are increased under hypoxic conditions [14], and increased glycolysis is associated with protection against oxidative stress [15]. Most glycolytic genes are transcriptionally regulated by hypoxia-inducible factor-1 (HIF1), a principal regulator of the molecular response to hypoxia in addition to PGAM [16,17].

Trans-activator of transcription (Tat), a protein transduction domain, was first discovered in human immunodeficiency virus-1 and shows great efficacy in penetrating the cell membrane and blood-brain barrier [18,19]. In a previous study, we synthesized a Tat-PGAM1 fusion protein to cross the blood-brain barrier and deliver PGAM1 intracellularly [20]. We also demonstrated that PGAM1 increased the energy utilization in the hippocampus of gerbils to protect neurons from ischemic damage [21]. In addition, PGAM1 increased hippocampal neurogenesis and phosphorylated cAMP response element binding protein in the dentate gyrus [20].

In the present study, we investigated the effects of Tat-PGAM1 and its control protein (Control-PGAM1) against oxidative stress in the NSC34 cell line, which is a hybrid of mouse neuroblastoma cells and motor neurons from the spinal cord of mouse embryos [22]. We also examined the effects of Tat-PGAM1 and Control-PGAM1 in rabbits to determine their neuroprotective potential against spinal cord ischemia.

## 2. Results

### 2.1. Tat-PGAM1, but Not Control-PGAM1, Was Effectively Delivered into NSC34 Cells

Tat-PGAM1 and Control-PGAM1 delivery was assessed using a western blot for polyhistidine with a His-Tag inserted into the vector to visualize the intracellular delivery of proteins. Polyhistidine bands were not observed at any concentration (0.5–5.0 μM) or time (15–60 min) after Control-PGAM1 treatment. In contrast, incubation with 1.0 μM Tat-PGAM1 significantly increased the polyhistidine levels in a concentration-dependent manner. In addition, incubation with 3.0 μM Tat-PGAM1 significantly increased the polyhistidine levels in a time-dependent manner by 60 min after treatment.

**Figure 1:**
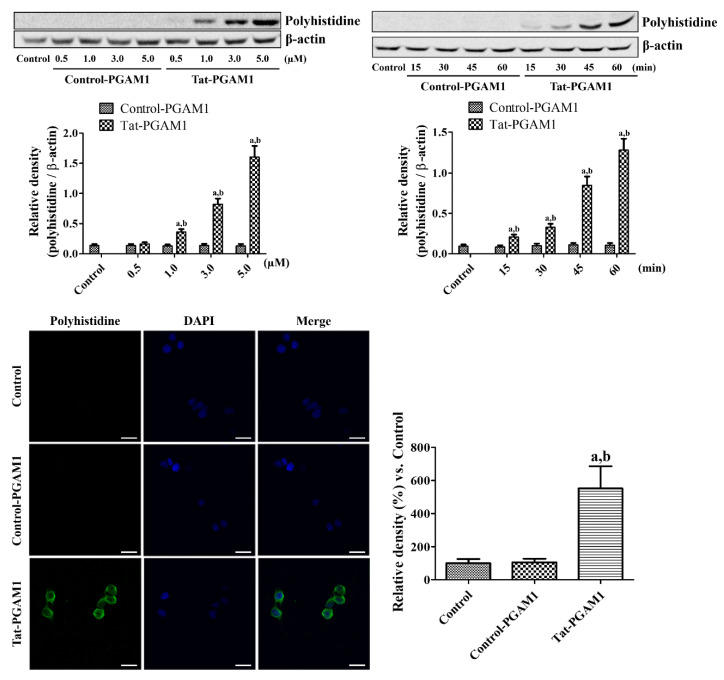
Intracellular delivery of Trans-activator of transcription-phosphoglycerate mutase 1 (Tat-PGAM1) and Control-PGAM1 into NSC34 cells. To assess the concentration dependence, NSC34 cells were incubated with various concentrations (0.5–5.0 μM) of Tat-PGAM1 and Control-PGAM1 proteins for 1 h. To assess the time dependence, NSC34 cells were incubated with 3 μM each of Tat-PGAM1 and Control-PGAM1 for various durations (15–60 min). Delivery was confirmed by western blot analysis for polyhistidines. The delivery of Tat-PGAM1 and Control-PGAM1 was visualized by immunohistochemical staining for polyhistidine 60 min after protein (both 3 μM) incubation. Scale bar = 50 μm. The optical densities of the polyhistidine bands and immunoreactivities were measured and the data were analyzed using two-way ANOVA followed by Bonferroni’s post hoc test (^a^*p* < 0.05, significantly different from the control group; ^b^*p* < 0.05, significantly different from the Control-PGAM1 group). The bar graph represents the mean ± standard deviation (SD).

Intracellular delivery of Tat-PGAM1 and Control-PGAM1 was visualized using immunocytochemical staining for polyhistidine 1 h after treatment with 3.0 μM Tat-PGAM1 and Control-PGAM1. In the control and Control-PGAM1-treated groups, no polyhistidine immunoreaction was seen in NSC34 cells. In the Tat-PGAM1-treated group, strong polyhistidine immunoreactivity was found in the cytoplasm, but not in the nucleus, of NSC34 cells. In addition, polyhistidine immunoreactivity was significantly increased in the Tat-PGAM1-treated group compared to that in the control and Control-PGAM1-treated groups (Figure 1).

### 2.2. Tat-PGAM1, but Not Control-PGAM1, Protects NSC34 Cells from Oxidative Damage

**Figure 2:**
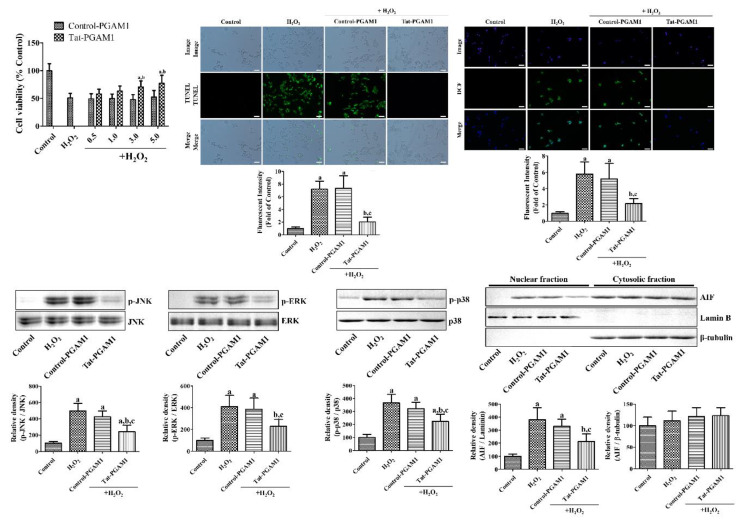
The protective effects of Tat-PGAM1 and Control-PGAM1 against oxidative damage in NSC34 cells. Oxidative stress was induced by treatment with 1 mM H_2_O_2_ for 3 h (terminal deoxynucleotidyl transferase-mediated biotinylated deoxyuridine triphosphate (dUTP) nick end labeling (TUNEL) staining), 5 h (water-soluble tetrazolium salt-1 (WST-1) assay), 10 min (2,7-dichlorofluorescein (DCF) fluorescence), or 6 h (western blot). The cells were incubated with Tat-PGAM1 and Control-PGAM1 (both 3 μM for TUNEL and DCF staining) for 60 min before treatment with 1 mM H_2_O_2_ and measured with each assay kit. The cell viability, DNA fragmentation, and reactive oxygen species (ROS) formation were measured using a WST-1 assay, TUNEL staining, and DCF fluorescence, respectively. Scale bar = 50 μm. Mitogen-activated protein kinase (MAPK) pathways were confirmed by western blot analysis for c-jun N-terminal kinase (JNK), extracellular-signal-regulated kinase (ERK), p38, and their phosphorylated antibodies. In addition, apoptosis inducing factor (AIF) levels were measured in nuclear and cytosolic fraction. The cell viability was measured and the data were analyzed using two-way ANOVA followed by Bonferroni’s post hoc test (^a^*p* < 0.05, significantly different from the control group; and ^b^*p* < 0.05, significantly different from the Control-PGAM1 group). The intensities of TUNEL positive cells, the DCF fluorescence, and the optical densities of the protein bands were measured and the data were analyzed using one-way ANOVA followed by Bonferroni’s post hoc test (^a^*p* < 0.05, significantly different from the control group; ^b^*p* < 0.05, significantly different from the only H_2_O_2_ group; and ^c^*p* < 0.05, significantly different from the Control-PGAM1 group). The bar graph represents the mean ± standard deviation (SD).

The cell viability and DNA fragmentation were measured using the water-soluble tetrazolium salt-1 (WST-1) assay and terminal deoxynucleotidyl transferase-mediated biotinylated deoxyuridine triphosphate (dUTP) nick end labeling (TUNEL) staining. Incubation with 1 mM H_2_O_2_ for 5 h dramatically decreased the cell viability to 50.9% of the control group. Pre-incubation with 0.5–5.0 μM Control-PGAM1 for 1 h and subsequent treatment with 1 mM H_2_O_2_ for 5 h had no significant effects on the cell viability at any concentration. However, incubation with Tat-PGAM1 increased the cell viability in a concentration-dependent manner; the cell viability was significantly increased in the 3.0 μM and 5.0 μM Tat-PGAM1-treated groups as compared to the only H_2_O_2_ group or the concentration-matched Control-PGAM1-treated group. In the 3.0 μM and 5.0 μM Tat-PGAM1-treated groups, cell viability was 70.7% and 77.6% of the control group, respectively (Figure 2).

In the control group, few TUNEL-positive cells were found in the NSC34 cell lines. Incubation with 1 mM H_2_O_2_ for 3 h showed several TUNEL-positive structures in the NSC34 cell line, and the fluorescence intensity was significantly higher (578.5%) in the control group. Pre-incubation with 3.0 μM Control-PGAM1 for 1 h and subsequent treatment with 1 mM H_2_O_2_ for 3 h showed several TUNEL-positive structures in NSC34 cells, and the fluorescence intensity was similar to the H_2_O_2_ alone group. Pre-incubation with 3.0 μM Tat-PGAM1 for 1 h and subsequent treatment with 1 mM H_2_O_2_ for 3 h dramatically decreased TUNEL-positive structures in NSC34 cells as compared to the H_2_O_2_ alone group, and the fluorescence intensity was 62.6% less than the H_2_O_2_ alone group, and 216.5% of the control group (Figure 2).

To elucidate the antioxidant effects of Tat-PGAM1 against H_2_O_2_-induced oxidative damage, the reactive oxygen species (ROS) levels were measured based on the 2,7-dichlorofluorescein (DCF) formation in NSC34 cells. In the control group, the DCF fluorescence levels in the NSC34 cells were very low; however, incubation with 1 mM H_2_O_2_ for 10 min and 20 μM DCF diacetate (DCF-DA) for 30 min significantly increased the DCF formation in NSC34 cells, and the fluorescence intensity was significantly increased to 721.9% of that of the control group. Sequential incubation with 3.0 μM Control-PGAM1 for 1 h, 1 mM H_2_O_2_ for 10 min, and 20 μM DCF-DA for 30 min showed abundant DCF formation in NSC34 cells. The DCF fluorescence intensity was similar to that of the H_2_O_2_ alone group. However, sequential incubation with 3.0 μM Tat-PGAM1 for 1 h, 1 mM H_2_O_2_ for 10 min, and 20 μM DCF-DA for 30 min significantly decreased the DCF formation in NSC34 cells as compared to the H_2_O_2_ alone group. In this group, the DCF fluorescence intensity was 71.6% less than the H_2_O_2_ alone group, and 205.0% of the control group (Figure 2).

The signal transduction mechanisms were assessed using western blot analysis for mitogen-activated protein kinases (MAPKs), such as c-Jun N-terminal kinase (JNK), extracellular-signal-regulated kinase (ERK), p38, and their phosphorylated forms (p-JNK, p-ERK, and p-p38, respectively). The levels of JNK, ERK, and p38 did not show any significant differences among groups. However, incubation with 1 mM H_2_O_2_ for 6 h showed significant increases of the p-JNK (497.0%), p-ERK (409.9%), and p-p38 (366.1%) protein levels compared to their control groups in NSC34 cells. Pre-incubation with 3.0 μM Control-PGAM1 for 1 h and subsequent treatment with 1 mM H_2_O_2_ for 6 h showed similar increases of the p-JNK, p-ERK, and p-p38 protein levels in the protein compared to those in control group, respectively. Pre-incubation with 3.0 μM Tat-PGAM1 for 1 h and subsequent treatment with 1 mM H_2_O_2_ for 6 h significantly decreased p-JNK (51.7% less than the H_2_O_2_ alone group, 240.1% of control), p-ERK (44.3% less than the H_2_O_2_ alone group, 228.5% of control), and p-p38 (39.0% less than the H_2_O_2_ alone group, 223.4% of the control) protein levels in NSC34 cells compared to those in Tat peptide treated group (Figure 2).

Apoptosis inducing factor (AIF) was also measured by western blot analysis in cytosolic and nuclear fractions of NSC34 cells. Incubation with 1 mM H_2_O_2_ for 6 h significantly increased AIF levels in the nuclear fraction by 379.9% of control group. Pre-incubation with 3.0 μM Tat-PGAM1, not Control-PGAM1, showed significantly lower levels of AIF (44.2% less than the H_2_O_2_ alone group, 212.1% of the control) in nuclear fraction than the H_2_O_2_ alone group. However, in the cytosolic fraction, the AIF levels did not show any significant differences among groups (Figure 2).

### 2.3. Tat-PGAM1, but Not Control-PGAM1, Prevents Neuronal Damage in the Ventral Horn of the Rabbit Spinal Cord

**Figure 3:**
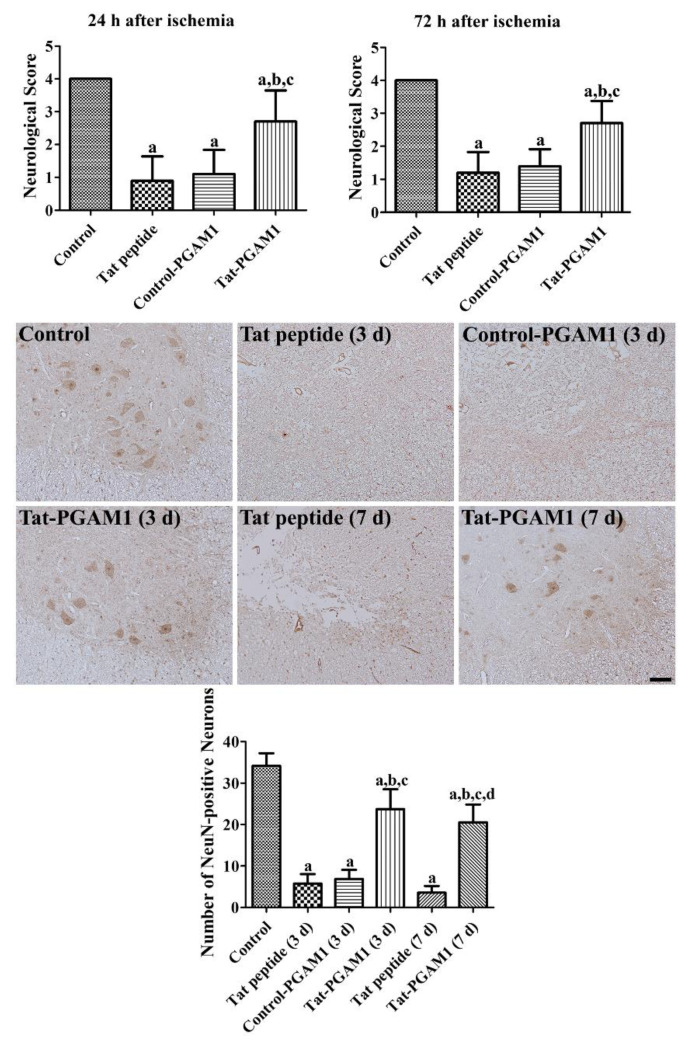
The protective effects of Tat-PGAM1 and Control-PGAM1 against ischemic damage in the rabbit spinal cord. Neurological scores of the control, Tat peptide-, Control-PGAM1-, and Tat-PGAM1-treated groups were measured using the modified Tarlov’s criteria at 24 and 72 h after ischemia/reperfusion. The animals were sacrificed 3 days or 7 days after ischemia for NeuN immunohistochemical staining in the lumbar segments (L_6_-L_7_) of the spinal cord. Scale bar = 100 μm. The number of NeuN-positive cells were counted in the spinal cord per section for all the groups, and the data were analyzed using one-way ANOVA followed by Bonferroni’s post hoc test (^a^*p* < 0.05, significantly different from the control group; ^b^*p* < 0.05, significantly different from the Tat peptide (3 d) group; ^c^*p* < 0.05, significantly different from the Control-PGAM1 (3 d) group; and ^d^*p* < 0.05, significantly different from the Tat peptide (7 d) group). Th bar graph represents the mean ± standard deviation (SD).

In the control uninjured group, the rabbits did not show any significant changes in the partial pressure of oxygen (PaO_2_), partial pressure of carbon dioxide (PaCO_2_), pH, and glucose before and 10 min after reperfusion (Table 1). Neurological changes induced by spinal cord ischemia were assessed by Tarlov’s neurological scores at 24 and 72 h after ischemia. In the control group, rabbits did not show any significant changes in their neurological scores at 24 or 72 h after ischemia, whereas in the Tat peptide-treated group, the neurological scores were significantly decreased when compared with the control group; they were 0.9 and 1.2 at 24 and 72 h after ischemia, respectively. In the Control-PGAM1-treated group, the neurological scores were similar to those of the Tat peptide treated group at 24 and 72 h after ischemia. The Tat-PGAM1-treated group showed significantly higher neurological scores than the Tat peptide- or Control-PGAM1-treated groups at 24 and 72 h after ischemia (Figure 3).

Neuronal damage was evaluated using neuronal nuclei (NeuN) immunohistochemical staining in the ventral horn of the spinal cord. In the control group, abundant NeuN-positive neurons were found in the spinal cord. In the Tat peptide- and Control-PGAM1-treated groups, a few NeuN-positive cells were observed in the ventral horn of the spinal cord, and this number was significantly decreased to 9.5% and 12.3% of the control group, respectively, 3 days after ischemia. In the Tat-PGAM1-treated group, some neurons were immunostained with NeuN, and this number was significantly increased to 65.0% of the control group when compared with the Tat peptide- and Control-PGAM1-treated groups 3 days after ischemia. Seven days after ischemia, fewer NeuN-positive cells were found in the Tat-peptide- (8.4% of control) or Tat-PGAM1-treated (57.1% of control) groups compared to those in 3 days post-ischemic groups. However, the number of NeuN-positive neurons were significantly higher in the Tat-PGAM1-treated group than that in the Tat-peptide-treated group (Figure 3).

### 2.4. Tat-PGAM1, but Not Control-PGAM1, Reduces Inflammatory Responses and Oxidative Damage after Ischemia

The inflammatory responses were evaluated using immunohistochemical staining for ionized calcium-binding adapter protein-1 (Iba-1) and enzyme-linked immunosorbent assay (ELISA) for interleukin (IL)-1β, IL-6, and tumor necrosis factor (TNF)-α. In the control group, Iba-1-immunoreactive microglia had low proportions of cytoplasm and long processes in the ventral horn of the spinal cord (inset image). In the Tat peptide-treated group, we observed numerous Iba-1-immunoreactive microglia with hypertrophied and round cytoplasm (phagocytic form, see the inset image). 

In this group, the Iba-1 immunoreactivity was significantly increased to 458.1% of the control group. In the Control-PGAM1-treated group, the Iba-1 immunoreactive microglia showed similar morphology, and the Iba-1 immunoreactivity was similar to that of the Tat peptide-treated group. In the Tat-PGAM1-treated group, the Iba-1 immunoreactive microglia had hypertrophied cytoplasm with retracted processes (activated form, inset image); however, phagocytic forms were rarely observed in the spinal cord. In this group, the Iba-1 immunoreactivity was significantly decreased compared to the Tat peptide- and Control-PGAM1-treated groups. The IL-1β in the Tat peptide- and Control-PGAM1-treated groups transiently increased at 24 h after ischemia and decreased 72 h after ischemia, when compared to the basal level. In the Tat-PGAM1-treated group, the IL-1β levels were significantly lower at 24 h and higher at 72 h after ischemia. Unlike IL-1β, the IL-6 and TNF-α levels in all groups gradually increased at 24 h and decreased at 72 h after ischemia. In the Tat-PGAM1-treated group, the IL-6 levels were significantly lower at 24 and 72 h after ischemia when compared to the time-matched Tat-peptide- and Control-PGAM1-treated groups. In addition, the TNF-α levels were significantly lower in the Tat-PGAM1-treated group than in the Tat-peptide- and Control-PGAM1-treated groups after ischemia.

**Figure 4:**
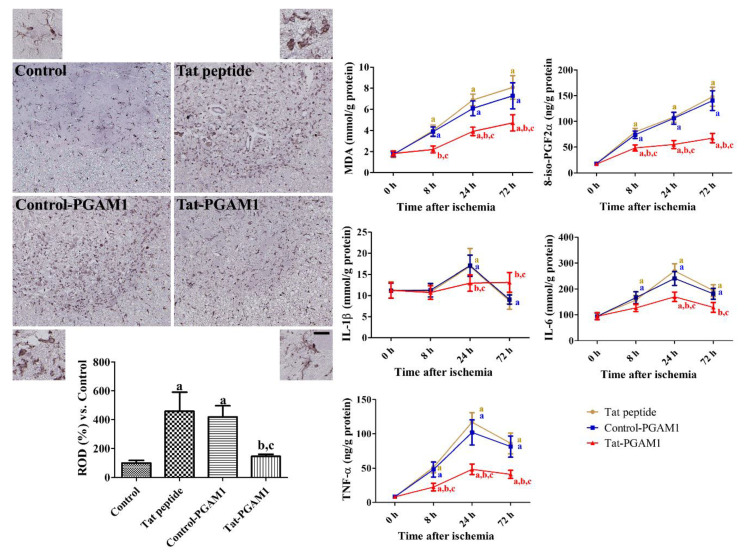
The anti-oxidative and anti-inflammatory effects of Tat-PGAM1 and Control-PGAM1 against ischemic damage in the rabbit spinal cord. Iba-1 immunohistochemistry was conducted to visualize the morphology of microglia/macrophages in the ventral horn of the spinal cord. Scale bar = 100 μm, 25 μm (inset image). The relative optical densities were expressed as percentile values of Iba-1 immunoreactivity versus control group per section. The lipid peroxidation and oxidized arachidonic acid were determined by MDA and 8-iso-PGF2α assay, respectively, after ischemia/reperfusion. In addition, pro-inflammatory cytokines, such as Interleukin (IL)-1β, IL-6, and tumor necrosis factor (TNF)-α were measured in the spinal cord after ischemia/reperfusion. The data were analyzed using one- or two-way ANOVA followed by Bonferroni’s post hoc test (^a^*p* < 0.05, significantly different from the control group; ^b^*p* < 0.05, significantly different from the Tat peptide group; and ^c^*p* < 0.05, significantly different from the Control-PGAM1 group). The bar graph represents the mean ± standard deviation (SD).

The oxidative damage was assessed by measuring the malondialdehyde (MDA) and 8-iso-prostaglandin F2α (8-iso-PGF2α) levels generated from the membrane at 8, 24, and 72 h after ischemia. In all groups, the MDA and 8-iso-PGF2α levels increased in a time-dependent manner in the spinal cord after ischemia. However, in the Tat-PGAM1-treated group, the MDA and 8-iso-PGF2α levels were significantly lower at 8, 24, and 72 h after ischemia compared to the time-matched Tat peptide- and Control-PGAM1-treated groups (Figure 4).

## 3. Discussion

PGAM is an enzyme in the glycolytic pathway that catalyzes the interconversion of 2- and 3-phosphoglycerate [10]. PGAM1 is highly susceptible to oxidative stress, which leads to the inhibition of glycolysis and is easily oxidized in conditions of neurological disease, such as Alzheimer’s disease and hypoxic damage [23,24,25]. In addition, PGAM1 levels are decreased in the brains of mice models of phenylketonuria [26] and in the hippocampus of mice exposed to copper toxicity [27]. In the present study, we synthesized a Tat-PGAM1 fusion protein, which easily crosses the blood–brain barrier and cell membrane, to elucidate its role in protecting against oxidative and ischemic damage in NSC34 cells and the rabbit spinal cord. We confirmed the intracellular delivery of the Tat-PGAM1 fusion protein into motor neuron-like cells, such as NSC34 cells in a concentration- and time-dependent manner. This result is consistent with a previous study showing that Tat-PGAM1 can be effectively delivered to hippocampal cell lines [21]. In addition, we observed the treatment with Tat-PGAM1 significantly reduced the neurological deficits induced by spinal cord ischemia 24 and 72 h after ischemia. NeuN immunohistochemistry releveled that Tat-PGAM1 protected neurons from ischemic damage in the spinal cord 72 h after ischemia, and we confirmed that this effect was maintained by 7 days after ischemia. We also observed that Tat-PGAM1, and not Control-PGAM1, protected neurons from oxidative damage; NSC34 cells incubated with 1 mM H_2_O_2_ showed reduced DNA fragmentation and ROS formation when treated with Tat-PGAM1.

H_2_O_2_ produced by oxidative stress activates MAPK [28,29], and the treatment with MAPK pathway inhibitor reduces neuronal damage in neural cells induced by H_2_O_2_ [30]. In the present study, we observed that 1 mM H_2_O_2_ significantly increased the phosphorylation of JNK, ERK, and p38 in NSC34 cells, and the treatment with Tat-PGAM1, not Control-PGAM1, significantly ameliorated the increases of MAPK phosphorylation in NSC34 cells exposed to H_2_O_2_. This result suggests that Tat-PGAM1 ameliorates the H_2_O_2_-induced activation of MAPKs, such as JNK, ERK, and p38 in the NSC34 cells. 

We also observed the nuclear fraction of AIF in NSC34 cells after exposure to H_2_O_2_ because the translocation of AIF into nucleus occurred in the cell death pathway [31]. Exposure to H_2_O_2_ significantly increased the nuclear AIF levels, and the treatment with Tat-PGAM1 ameliorated the translocation of AIF, indicating the protective potentials of Tat-PGAM1. This result is supported by previous studies that showed that enhanced glycolytic flux decreased cellular senescence by reducing oxidative damage [13,32].

In addition, we examined MDA and 8-iso-PGF2α, which are indicators of the oxidative status [33]. Spinal cord ischemia significantly increased the MDA and 8-iso-PGF2α levels in the spinal cord, and these results are consistent with previous studies that showed that spinal cord ischemia increased biomarkers of free radical damage [5,8,34]. Treatment with Tat-PGAM1, and not Control-PGAM1, significantly ameliorated the increase in MDA and 8-iso-PGF2α levels in the spinal cord. These results suggest that Control-PGAM1 could not cross the blood-brain barrier or cell membrane in the spinal cord and that Tat-PGAM1 reduces lipid peroxidation and oxidized arachidonic acid products induced by ROS after spinal cord ischemia. PGAM1 is known to reduce oxidative stress by inactivation of nicotinamide adenine dinucleotide phosphate under hypoxic conditions [35].

Neuroinflammation is closely related to the energy metabolism. Pro-inflammatory macrophages receive energy from glycolysis, whereas anti-inflammatory macrophages rely on oxidative phosphorylation for their function [36,37,38]. In the present study, we observed Iba-1-immunoreactive microglia and pro-inflammatory cytokine levels in the spinal cord because pro-inflammatory cytokines are known to play important roles in hyperalgesia and the death of neuronal and glial cells in association with secondary spinal cord injuries [39,40,41,42,43]. Transient forebrain ischemia significantly changes the morphology of Iba-1-immunoreactive microglia, increasing the proportion of phagocytic microglia to remove cells that died due to ischemia damage [44]. Treatment with Tat-PGAM1 significantly reduced the phagocytic form of microglia, indicating reduced inflammatory responses in the spinal cord after ischemia as compared to the Tat peptide- and Control-PGAM1-treated groups.

Several studies have demonstrated glial activation and crosstalk with pro-inflammatory cytokines [27,45] to reduce the propagation of damage after ischemia. In the present study, we observed transient increases in pro-inflammatory cytokines, such as IL-1β, IL-6, and TNF-α in the spinal cord after ischemia, indicating inflammation. TNF-α and IL-1β levels increase after spinal cord injury [7] and biphasically peak at 6 and 36–48 h after ischemia/reperfusion [6]. However, IL-6 is transiently increased and returns to basal levels 4 days after ischemia [46,47]. Treatment with Tat-PGAM1 significantly alleviated the transient increases in pro-inflammatory cytokines after ischemia and reduced the inflammatory response. However, in immature murine articular chondrocytes, transfection of PGAM1 increased IL-1β, IL-6, and TNF-α transcription levels in a concentration-dependent manner [48]. This contradictory result may be associated with specific conditions in the animal model and target organs. In the present study, we compared IL-1β, IL-6, and TNF-α levels in the spinal cord after ischemia, whereas Song et al. measured cytokine levels in immature articular chondrocytes under normal conditions [48].

Collectively, our study investigated the effects of Tat-PGAM1 on H_2_O_2_-induced oxidative stress in NSC34 cells and ischemia-induced cell damage. Tat-PGAM1 lowers the ROS formation, MAPK activation, and nuclear translocation of AIF in NSC34 cells and membrane peroxidation in the spinal cord. In addition, Tat-PGAM1 reduces the microglial activation and subsequent increases in pro-inflammatory cytokines after ischemia. These results suggest that Tat-PGAM1 could reduce the complications induced by ischemic damage in the rabbit spinal cord.

## 4. Materials and Methods

### 4.1. Concentration- and Time-Dependent Delivery of Tat-PGAM1 into NSC34 Cells

As described in a previous study [20], Tat-PGAM1 and Control-PGAM1 proteins were generated with and without Tat, respectively, based on a human PGAM1 cDNA clone in a TA vector. To differentiate intracellular PGAM1 from exogenous Tat-PGAM1 and Control-PGAM1, a His-tag was inserted into the vector system. Tat-PGAM1 or Control-PGAM1 plasmids were transformed into *Escherichia coli* BL21 cells, cultivated in broth media, and purified using a Ni^2+^-nitrilotriacetic acid Sepharose affinity column (Qiagen, Chatsworth, CA, USA) and PD-10 column chromatography (GE Healthcare, Chicago, IL, USA).

Concentration- and time-dependent delivery of Tat-PGAM1 and Control-PGAM1 was observed using western blotting for polyhistidines. Briefly, NSC34 cells were exposed to various concentrations (0.5–5.0 μM) of Tat-PGAM1 or Control-PGAM1 protein for 60 min or to 3 μM protein for various periods (15–60 min).

### 4.2. Visualization of Intracellular Delivery of Tat-PGAM1 in NSC34 Cells

The intracellular delivery of Tat-PGAM1 and Control-PGAM1 was visualized using immunocytochemical staining for polyhistidine. Briefly, coverslip-grown NSC34 cells were exposed to 3 μM Tat-PGAM1 or Control-PGAM1 protein for 60 min and the cells were fixed with 4% paraformaldehyde (PFA) in 0.1 M phosphate buffer (PB, pH 7.4) for 5 min at 25 °C. Thereafter, the cells were sequentially incubated with mouse anti-polyhistidine antibody (Sigma, St. Louis, MO, USA) and Alexa Fluor^®^ 488-conjugated anti-mouse Immunoglobulin G (1:1000; Jackson ImmunoResearch, West Grove, PA, USA) with 1 μg/mL 4,6-diamidino-2-phenylindole (DAPI; Thermo Fisher Scientific, Waltham, MA, USA). The immunoreaction was observed under a confocal fluorescence microscope (LSM 510 META NLO; Zeiss GmbH, Jena, Germany) and the fluorescence was measured using a Fluoroskan ELISA plate reader (Labsystems Oy, Helsinki, Finland) at 485 nm excitation and 538 nm emission.

### 4.3. Neuroprotective Effects of Tat-PGAM1 against Oxidative Damage in NSC34 Cells

Oxidative stress in NSC34 cells was induced by treatment with 1 mM H_2_O_2_, and the neuroprotective effects of Tat-PGAM1 and Control-PGAM1 were assessed using WST-1 and TUNEL assays, as described in a previous study [49]. Briefly, various concentrations (0.5–5.0 µM) or 3.0 µM of Tat-PGAM1 and Control-PGAM1 were added to NSC34 cells for 60 min, followed by 1 mM H_2_O_2_, and then incubated for 3 h or 5 h for TUNEL and WST-1 assays, respectively. The TUNEL staining and WST-1 assay were conducted as per the manufacturer instructions. The cell viability was assessed using the WST-1 assay, by measuring optical density at 450 nm using an ELISA microplate reader (Labsystems Multiskan MCC/340, Helsinki, Finland). DNA fragmentation by TUNEL staining was visualized using a fluorescence microscope (Nikon Eclipse 80i, Tokyo, Japan) and fluorescence was measured using a Fluoroskan ELISA plate reader (Labsystems Oy) at 485 nm excitation and 538 nm emission.

The intracellular ROS levels were visualized by monitoring the DCF formation from DCF-DA as described previously [8,49]. Briefly, 3.0 µM of Tat-PGAM1 and Control-PGAM1 were added to NSC34 cells for 60 min, followed by 1 mM H_2_O_2_, and then incubated for 10 min. The cells were incubated with 20 μM DCF-DA for 30 min, and the fluorescence levels were measured using a Fluoroskan ELISA plate reader (Labsystems Oy) at 485 nm excitation and 538 nm emission.

The MAPK and AIF levels were assessed by western blot analysis using antibodies for JNK (1:500, Cell Signaling Technology, Inc., Beverly, MA, USA), ERK (1:500, Cell Signaling Technology, Inc.), p38 (1:1000, Cell Signaling Technology, Inc.), p-JNK (1:200, Cell Signaling Technology, Inc.), p-ERK (1:200, Cell Signaling Technology, Inc.), p-p38 (1:500, Cell Signaling Technology, Inc.), AIF (1:200, Cell Signaling Technology, Inc.), Laminin (1:1000, SantaCruz Biotechnology, Santa Cruz, CA, USA), and β-tubulin (1:1000, SantaCruz Biotechnology). Briefly, 3.0 µM of Tat-PGAM1 and Control-PGAM1 were added to NSC34 cells for 60 min, followed by 1 mM H_2_O_2_, followed by 1 mM H_2_O_2_, and then incubated for 6 h.

### 4.4. Neuroprotective Effects of Tat-PGAM1 against Ischemic Damage in Rabbits

Male New Zealand white rabbits (1.2–1.5 kg) were used in the study. Animals were supplied by DooYeol Biotech (Seoul, Korea). The experimental protocol was approved by the Institutional Animal Care and Use Committee (IACUC) of Seoul National University (approval number: SNU-191017-21). Animals were anesthetized with 2.5% isoflurane (Hana Pharm Co., Ltd., Hwaseong, Korea) with a mixture of 67% N_2_O and 33% O_2_ gas. A midline incision was made in the abdominal region and the abdominal aorta was exposed. The abdominal aorta was occluded in the subrenal region using non-traumatic aneurysm clips for 30 min, and reperfusion was confirmed by observation under a stereoscope. The body temperature was tightly regulated (38.7 ± 0.3 °C) during ischemic surgery using a thermometric blanket connected to a rectal probe. Immediately after reperfusion, the rabbits received an intraperitoneal injection of Tat peptide (2.5 mg/kg), Control-PGAM1 protein, or Tat-PGAM1 protein (both 2.5 mg/kg). The dosage of Tat-PGAM1 was determined by previous studies showing the neuroprotective effects of Tat-PGAM1 against ischemic damage [21] and translation of dosage (10 mg/kg × 3/12) [50].

Physiological parameters, such as PaO_2_, PaCO_2_, pH, and glucose were analyzed using a GEM Premier 3000 (Instrumentation Laboratory, Milan, Italy) before and 10 min after reperfusion. In addition, the mean arterial pressure (MAP) was measured from the coccygeal artery. The neurological status was evaluated based on the Tarlov criteria [51] 24 and 72 h after reperfusion as described in previous studies [8,49], because postural abnormalities were found 12–24 h after ischemia; complete paraplegia was seen 48 h after ischemia [52,53].

To observe the neuroprotective effects of Tat-PGAM1 and Control-PGAM1 against ischemic damage, the surviving neurons and microglia were visualized using immunohistochemical staining for NeuN and Iba-1 in the ventral horn of the spinal cord, respectively, as described in a previous study [54]. The rabbits were re-anesthetized with 5% isoflurane 3 and 7 d after reperfusion and perfused transcardially with physiological saline and 4% PFA. Lumbar spinal cord (L_6_–L_7_) was removed from the vertebral column and post-fixed in the same fixative for 24 h. Spinal tissue was dehydrated with ethanol, cleared with xylene, and embedded in paraffin wax. Coronal sections (4 μm thickness) were prepared and sections were incubated with mouse anti-NeuN (1:1000; EMD Milipore, Temecula, CA, USA) and anti-Iba-1 (1:100; Abcam, Cambridge, UK) for 48 h at 4 °C. Thereafter, sections were sequentially incubated with goat anti-mouse IgG and a streptavidin-peroxidase complex (1:200; Vector, Burlingame, CA, USA) for 2 h at 25 °C. Sections were visualized by reaction with 3,3′-diaminobenzidine tetrachloride (Sigma), hydrated with ethanol, and mounted with Canada balsam (Junsei. Chemical, Tokyo, Japan).

### 4.5. Anti-Oxidative and Anti-Inflammatory Effects of Tat-PGAM1 Against Ischemic Damage in Rabbits

Oxidative damage induced by spinal cord ischemia was assessed by measuring MDA and 8-iso-PGF2α, which are generated by the peroxidation of polyunsaturated fatty acid and arachidonic acid in the membrane, respectively [55,56]. The inflammatory response induced by spinal cord ischemia was evaluated by measuring pro-inflammatory cytokines, such as IL-1β, IL-6, and TNF-α. Briefly, animals (*n* = 5 per group) were anesthetized using 5% isoflurane, and the lumbar spinal cords (L_6_–L_7_) were obtained. MDA (Cayman Chemical Company, Ann Arbor, MI, USA), 8-iso-PGF2α (Cayman Chemical Company), IL-1β (Cusabio, Hubei, China), IL-6 (Cusabio), and TNF-α (R&D Systems Inc., Minneapolis, MN, USA) ELISA assay kits were used to measure the oxidative indicators and pro-inflammatory cytokines as per the manufacturer instructions.

### 4.6. Data Quantification and Analysis

The number of NeuN-positive cells was counted in the ventral horn of the spinal cord using Optimas 6.5 image analysis software (CyberMetrics Corporation, Phoenix, AZ, USA). Ten sections were analyzed to count NeuN-positive cells in the spinal cord, and the average number of NeuN-positive cells was noted.

The Iba-1 immunoreactivity was analyzed using ImageJ software version 1.50 (National Institutes of Health, Bethesda, MD, USA), as described previously [20,21]. Images taken from Iba-1 immunoreactive structures were calibrated to 256 gray scale, and unlabeled structures were subtracted using Adobe Photoshop 2020 software. The pixel number and immunodensity were measured using ImageJ, and the optical density of Iba-1-immunoreactive structures was calculated. All optical densities were represented as percentile values versus the control group (set as 100%).

All data shown are presented as the mean ± standard deviation. Differences in the means were compared and statistically analyzed using one-way or two-way analysis of variance (ANOVA), followed by Bonferroni’s post hoc test using GraphPad Prism 5.01 software (GraphPad Software, Inc., La Jolla, CA, USA).

## Figures and Tables

**Table 1:** The physiological parameters before and 10 min after spinal cord ischemia in rabbits.

	pH	Distal Mean Arterial Pressure (MAP) (mmHg)	PaCO_2_ (mmHg)	PaO_2_ (mmHg)	Glucose (mM)
Pre-ischemia					
Control	7.39 ± 0.027	83.8 ± 9.14	37.2 ± 4.13	104.6 ± 9.15	6.43 ± 0.98
Vehicle	7.42 ± 0.033	84.2 ± 8.81	36.9 ± 3.80	103.5 ± 8.72	6.52 ± 1.29
Control-PGAM1	7.41 ± 0.035	84.1 ± 9.42	37.1 ± 3.75	106.4 ± 11.3	6.38 ± 0.81
Tat-PGAM1	7.39 ± 0.032	84.0 ± 8.06	36.8 ± 4.37	103.9 ± 10.4	6.41 ± 1.05
Reperfusion 10 min					
Control	7.41 ± 0.032	84.1 ± 8.60	37.0 ± 4.42	103.7 ± 9.71	6.37 ± 1.08
Vehicle	7.35 ± 0.068	88.7 ± 11.3	39.3 ± 5.95	111.4 ± 11.9	7.10 ± 1.42
Control-PGAM1	7.36 ± 0.092	87.8 ± 10.2	39.7 ± 5.28	106.6 ± 12.7	6.83 ± 1.27
Tat-PGAM1	7.37 ± 0.088	86.3 ± 9.92	38.1 ± 5.60	114.1 ± 11.1	7.25 ± 1.62

Transient spinal cord ischemia had no significant effects on physiological parameters in the control, vehicle-treated, Control-PGAM1-treated, and Tat-PGAM1-treated groups.
